# Trigeminal nociceptive somatotopy across brainstem and cortex and their functional interactions

**DOI:** 10.1186/s10194-026-02476-y

**Published:** 2026-08-31

**Authors:** Adrian Scutelnic, Kuan-Po Peng, Kristina Kokian, Arne May, Jan Mehnert

**Affiliations:** 1 Department of Systems Neuroscience, University Medical Center Hamburg-Eppendorf, Hamburg, Germany; 2 Department of Neurology, Bern University Hospital, Inselspital, University of Bern, Bern, Switzerland

## Abstract

**Background:**

The trigeminal nerve innervates the face and the meninges via its three branches, with central relay neurons located in the brainstem’s spinal trigeminal nucleus. We have shown a somatotopic organization on human brainstem level and aimed, using an independent new sample, to characterize the functional somatotopy with a focus on within-brainstem and brainstem–cortical connectivity.

**Methods:**

Electrical stimulation was applied to the three branches of the trigeminal nerve at two intensities (high and low) to examine the somatotopic organization in healthy participants during fMRI. Neuroimaging was preregistered (NCT06534880) and covered the upper spinal cord, brainstem, thalamus, insula, primary somatosensory cortex (S1), S2/parietal operculum, and cingulate cortex. Activation patterns following stimulation of each trigeminal branch were analyzed. Branch-specific functional connectivity was estimated within the brainstem as well as between brainstem and cortical targets.

**Results:**

In a cohort of 30 healthy participants (mean age 32.1 ± 8.8 years, 18 female (60%)), spatially distinct brainstem representations of the trigeminal branches were identified, replicating previous reports. Activation at the cortical level was largely bilateral and symmetric, except in the primary somatosensory cortex and the insula, where it was predominantly contralateral. Within the brainstem, functional connectivity was strongest between the left and right subnuclei at the same level and was independent of the stimulated branch (all F(2,116) ≤ 5.67, threshold F = 8.00, α = 0.05/90). Brainstem-cortex connectivity showed a robust rostrocaudal gradient that was modulated by the stimulated branch across insular, somatosensory, and opercular regions (significant in 37/48 ROIs, F-Range 2.46–5.19, P_FWE_<0.05).

**Conclusion:**

We replicated somatotopic representation of the three trigeminal branches in the brainstem and revealed distinct bilateral activation and branch-specific functional connectivity across multiple levels of the afferent neuraxis.

**Supplementary Information:**

The online version contains supplementary material available at https://doi.org/10.1186/s10194-026-02476-y.

## Introduction

The three branches of the trigeminal nerve (V1–V3) innervate distinct facial territories and the meninges, and are central to facial pain and headache disorders [1, 2]. Because the trigeminal nerve’s first central relay lies in the brainstem (the spinal trigeminal nucleus, STN), the entire nociceptive pathway up to the cortex fits within a single fMRI field of view — an advantage not available for spinal somatic nociception [3–7]. Despite the extensive characterization of STN anatomy, physiology, and connectivity in animals [8–10], direct functional neuroanatomy in humans remains sparse. Traditional anatomical data derive largely from post-mortem studies, while in-vivo characterization of trigeminal functional representations and their somatotopy has only recently become feasible with advances in high-resolution fMRI [11, 12]. Based on animal studies, an intermingled onion-shaped somatotopy of facial representation of trigeminal nociception has been postulated for the human STN [13, 14] and recently shown in fMRI studies optimized for the brainstem from our group [4]. Notably, this earlier work identified not one but multiple levels of representations of trigeminal input within the brainstem, which largely overlapped between the individual branches and the greater occipital nerve [4]. As the functional significance of these multiple representations remains unclear, and since the cortical representation could not be assessed due to the limited field of view in the brainstem-optimized study [4], we used an improved MRI protocol that covers the brainstem and the cortex in a new and independent cohort, and focused on whether the functional connectivity between brainstem and cortical target regions can distinguish the three trigeminal branches.

## Methods

### Study population and experimental design

This study was approved by the local ethics committee (Ethikkommission der Ärztekammer Hamburg, PV 5490), was preregistered (clinicaltrials.gov: NCT06535880 on 30 July 2024) and was conducted in accordance with the declaration of Helsinki. Written informed consent was obtained from all participants. Healthy individuals aged 18–65 years without contraindication for MRI were included. Exclusion criteria were: (i) known primary headache disorder, except for infrequent episodic tension-type headache with < 12 episodes per year, (ii) primary headache disorders in first-degree relatives, (iii) other psychiatric, neurologic, or systemic disorders; (iv) intake of any painkiller for more than 14 days a month, (v) full beard or skin disorders hindering the attachment of the electrodes, and (vi) non-German speaker.

### Experimental design: electrode positioning and equipment

**Fig. 1:**
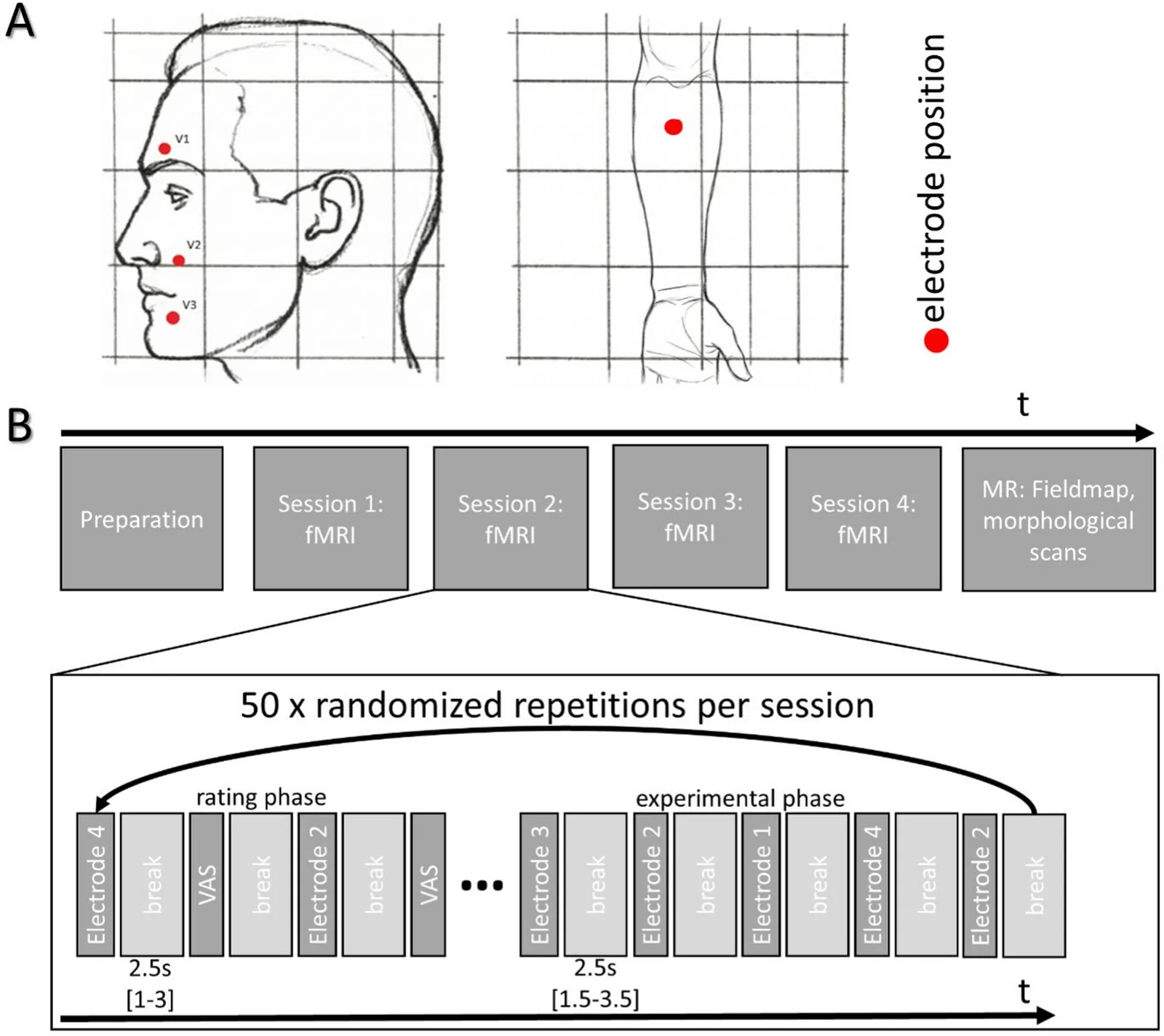
Experimental Design. (**a**) Schematic representation of the experimental design. (**b**) Timeline of the experiments. EDT, electrical detection threshold; VAS, Visual Analogue Scale; V1, opthtalmic division of the trigeminal nerve; V2, maxillar division of the trigeminal nerve; V3, mandibular division of the trigeminal nerve

This experiment consisted of repetitive electrical stimulation using an MR-compatible Digitimer DS7A (Digitimer Ltd., Welwyn Garten City, UK) with four shielded electrodes (Specialty Developments, Bexley, UK) using a D188 Remote Electrode Selector (Digitimer Ltd., Welwyn Garden City, UK) and custom-build MR-compatible cables. The cables were assembled using an MR-safety design to avoid tissue damage due to currents induced by electromagnetic wave coupling. The four electrodes were attached while the participant was seated on the MR-table. The electrodes were placed as follows: the V1 electrode on a vertical line through the middle of the left eyebrow, approximately 1 cm above it; the V2 electrode 1 cm lateral on a horizontal line passing through the inferior border of the left nostril; the V3 electrode on a vertical line passing through the left corner of the mouth, 1 cm inferior to it; and lastly, the arm electrode was placed on the left forearm, 5 cm below the cubital crease on the volar side (Fig. 1).

After placing the electrodes, the subjects were moved into the scanner. As a first step, the electrical pain thresholds (EPT) were assessed using the Quick Estimation by Sequential Testing (QUEST) procedure at the stimulation sites while manipulating the delivered current at the third division of the trigeminal nerve [15, 16]. The intensity used for higher sensory input was set to three time this pain threshold, while the intensity for the lower condition was set to 80% of the EPT. The experiment was divided into 4 blocks, where the higher intensity was delivered in two randomly assigned ones and the lower in the remaining two. Within each block, participants received one stimulus at each stimulation site, followed by a rating on the visual analog scale (VAS). Stimulus duration (2ms) was identical across all conditions and stimulation sites. After the rating phase, 50 stimuli were delivered to each location in randomized order. The participants therefore received a total of 200 trials per stimulation site (100 low, 100 high) over approximately 40 min of fMRI scanning.

### Primary and secondary outcomes

As preregistered, the primary outcome was the electrical nociception-induced activation in the field of view of all previously shown representations of nociceptive input for all three trigeminal branches. An additional primary outcome was the activation of the insula, primary and secondary somatosensory areas, and anterior cingulate cortex. The secondary outcome was the functional connectivity between each of the trigeminal brainstem representations as well as of each brainstem representation with preassigned higher cortical areas.

### MRI data acquisition and processing

For the MRI examinations a Siemens 3-T PRISMA scanner (Siemens, Erlangen) with a 64-channel head coil was used. First, fieldmaps were recorded (repetition time 0.858 s, echo times 7.97 ms and 5.51 ms, 3 × 3 × 2.5 mm^3^ spatial resolution, flip angle 20^o^, 78 slices, FOV 222 mm, no gap) covering the same volume as the following EPIs to attenuate inhomogeneities of the magnetic field. During the experiment we recorded four sessions with 284 volumes each using an EPI protocol (repetition time 2.116 s, echo time 33 ms, 1.25 × 1.25 × 2.50 mm^3^ spatial resolution, GRAPPA acceleration, flip angle 70^o^, 78 slices with a multiband factor of 2, FOV 215 mm, no gap, flow rephasing) with an FOV covering the brainstem as low as C2/3, cerebellum, midbrain, and the whole cortex. Pulse and breathing were recorded simultaneously to attenuate extracerebral (i.e. cardiovascular and pulmonary) artifacts. Last, we acquired high resolution anatomical images (MPRAGE, repetition time 7.1 ms, echo time 2.98 ms, 1 × 1 × 1 mm^3^, flip angle 9^o^, 240 slices, FOV 256 mm).

All fMRI data were filtered using the spatially adaptive non-local means filter implemented in the CAT12 toolbox [17]. The fMRI data were then corrected for movements and for distortions of the homogeneity of the magnetic field (fieldmaps) using the realign and unwarp algorithm as implemented in SPM12. Additionally, slice time correction was done using the onsets of the single slices adapted to our multiband protocol. A subject-wise general linear model (GLM) including condition-wise onsets of each stimulus as stick functions, which were then convolved with a hemodynamic response function (HRF). The button box responses as well as the onset and duration of the VAS were modelled as regressors of no interest. Additional regressors of no interest were included to correct for uncorrelated movements and cardiovascular influence using the algorithms proposed by Deckers et al. and for changes in the spinal fluid extracted from the fourth ventricle [18]. Co-registered structural images were segmented using the unified segmentation approach algorithm implemented in SPM12. For this process, we used templates provided by Blaiotta et al., which are optimized for the brainstem and spinal cord [19, 20]. This step generated the deformation fields used to warp the contrast images from the subject-level GLM into Montreal Neurological Institute (MNI) space. Each step was controlled by visual inspection. A group template was calculated, as well as gray and white matter masks from the warped structural images.

In the first-level analysis, input of each branch was averaged across sessions. In the second level analysis, we inferred the individual stimulus intensities for each branch and session by using the individual’s intensity ratings as to create a weighted contrast between high and low intensive sessions. Sessions with no perceived sensation were excluded due to potentially poor contact of the electrodes. Resulting statistical parametric maps were smoothed with an isometric Gaussian kernel of 4 mm^3^ and fed into group statistics by means of repeated measure ANOVAs.

### Generalized psychophysiological interaction

Functional connectivity was assessed by generalized psychophysiological interaction (gPPI) analyses using the gPPI toolbox (v13.1; McLaren et al., 2012, https://www.nitrc.org/projects/gppi). For each participant and each seed region, the gPPI toolbox extracted the first eigenvariate of the seed time series and computed interaction terms between the physiological signal and each experimental site (V1, V2 and V3). Two gPPI analyses were performed. Following our preregistration as well as our previous publication (Table 2 in [4]), we first used five seeds placed on the midline at each brainstem level — below pons (z = − 56), lower cerebellum (z = − 66), C1 (z = − 78), C2 (z = − 84) and C2/C3 (z = − 93). Two sets of brainstem seeds were used for the connectivity analyses. (i) For the cortico-brainstem connectivity analysis, fifteen seeds were defined (three trigeminal branches × five brainstem/spinal levels; Table 2 in [4]), each a 4 mm radius sphere centered on the midline (x = 0) at the a priori reference coordinates of the corresponding branch and level, which were taken from our previous work rather than from the present activation peaks to avoid circularity. (ii) For the lateralized within-brainstem connectivity analysis, ten seeds were defined (five levels × two hemispheres) by subdividing the brainstem mask into five contiguous, non-overlapping axial bands corresponding to the below pons (z = − 61 to − 51), lower cerebellum (z = − 72 to − 61), C1 (z = − 81 to − 72), C2 (z = − 88 to − 81), and C2/C3 (z = − 98 to − 88), and splitting each band at the midline (x = 0) into a left (x < 0) and a right (x > 0) portion. These seeds were anatomical half-masks and shared no voxels between hemispheres. For both seed sets, each MNI-defined seed was transformed into every participant’s native space via the inverse deformation field, mapping each voxel individually rather than only the center coordinate; the first eigenvariate of each native-space seed was then extracted for the gPPI models. At the group level, the resulting contrast images for each site-specific connectivity estimate were smoothed with a 4 mm FWHM Gaussian kernel. Group-level inference was performed using within-subject ANOVAs (*n* = 30) for each seed regions, as well as a two-factor flexible factorial ANOVA with stimulation branch (V1, V2, V3) and brainstem level as within-subject factors to test for main effects and their interaction. Statistical maps were thresholded using family-wise error correction (P_FWE_ < 0.05). Small-volume correction was applied within preregistered, anatomically pre-defined cortical target regions (insula subregions, S1, S2/parietal operculum, cingulate cortex), each defined as a 6 mm radius sphere centered on coordinates derived from the JuBrain Cytoarchitectonic Atlas v3.0 [21].

### Statistical analyses

#### Behavioral data analysis

Perceived pain intensity ratings were analyzed using a linear mixed-effects model with stimulus location (V1, V2, V3, arm) and stimulus intensity (high, low) as fixed effects, and a random intercept for subject. Sessions in which any stimulation received a rating of 1 (no perceived sensation) were excluded, resulting in an unbalanced design that was accommodated by the random-intercept structure. Degrees of freedom were approximated using the Satterthwaite method, and Type III ANOVA was used to test the significance of each fixed effect.

#### fMRI data analysis

At the group level we ran four second-level analyses, each addressing a different aspect of the data. (i) Unweighted ANOVA (*n* = 30) — our main estimate of site-specific activation, using contrast images averaged across all sessions. (ii) Rating-weighted ANOVA (*n* = 30) — to account for the between-subject variability in perceived pain, we scaled each participant’s contrast weights by their mean pain rating for that site (normalized to sum to one across participants). This ensures that participants with clearer intensity discrimination contribute more to the group estimate, reducing the influence of participants whose ratings showed little differentiation between intensity levels. (iii) Intensity-contrast ANOVA (*n* = 27) — an 8-condition ANOVA (4 sites × 2 intensity levels) restricted to sessions where V1 and arm ratings differed by 15 points or less, used to compare the intensity effect (high > low) between trigeminal and arm stimulation. (iv) Matched-trigeminal sensitivity ANOVA (*n* = 23) — a version restricted to sessions where all pairwise trigeminal branch ratings differed by 15 points or less, used for reliability analysis as it offers the possibility to depict cortical somatotopical arrangements of the trigeminal branches by a winner-take-all approach [11, 12].

All maps were thresholded at peak-level pFWE < 0.05 with small-volume correction. For the brainstem, we used 6 mm spheres around the reference coordinates from Mehnert et al. (2023) [4]. For the cortex, masks were built from the Jülich-Brain Atlas v3.0 and covered the insula (Ig1–Ig3 posterior, Id2–Id3 intermediate, Id4–Id7 anterior) [22, 23], S1 (Areas 3a, 3b, 1, 2) [24–26], S2/parietal operculum (OP1–OP4) [27, 28] and the cingulate cortex (Area 25, Area 33, s32, p32, p24ab, p24c, s24) [29, 30], all bilateral (24 subregions per hemisphere) [21]. The peak-level FWE threshold was computed per mask with SPM’s random field theory. For the somatotopy analyses, the trigeminal branch with the highest T-value at each suprathreshold voxel was assigned as the dominant branch (winner-take-all) [11, 12].

*gPPI.* For the gPPI region-of-interest analysis, we defined 24 cortical ROIs based on the Jülich-Brain Atlas v3.0: the anterior (Id4–Id7), intermediate (Id2–Id3), and posterior (Ig1–Ig3) insular subregions, the primary somatosensory cortex (Areas 3a, 3b, 1, 2), S2/parietal operculum (OP1–OP4), and the cingulate cortex (Area 33, p24c, p24ab, s24, p32, s32, Area 25) [21]. Additionally, we included the thalamus as a region of interest and defined it using the Morel thalamus atlas [31]. For each ROI, peak F-values were extracted within a 6 mm radius sphere from the voxelwise flexible factorial ANOVA (stimulation branch × brainstem level), and small-volume corrected p-values were computed using random field theory. To account for multiple comparisons, Bonferroni correction was applied separately within each a priori defined anatomical region, treating left and right hemisphere ROIs as independent tests.

#### Power calculation

Sample size was determined based on previous results [4]. The smallest significant effect in the brainstem activation (T = 3.58, *n* = 61) corresponded to an effect size of d ≈ 0.46. A power analysis indicated that a sample of 31 participants would be required to detect an effect of this magnitude with a statistical power of at least 0.80, using a one-sided test with a Type I error rate of α = 0.05.

### Data availability statement

Access to anonymized data and access to the code are available to qualified investigators on request to the corresponding author.

## Results

### Participants

We recruited 34 participants, of whom four were excluded: two due to panic attacks during scanning, one due to technical failure of the stimulation electrode, and one due to an incomplete dataset. The final sample comprised 30 participants (mean age 32.1 ± 8.8 years, 18 (60%) female).

### Behavioral data

Individual pain thresholds were determined for each participant (mean = 1.47 mA, SD = 1.07, range: 0.24–3.48). Low and high stimulation intensities were set at 0.8× (mean = 1.18 mA, SD = 0.86) and 3× (mean = 4.18 mA, SD = 3.00) the individual threshold, respectively (Table 1). High-intensity stimulation yielded significantly higher pain ratings than low-intensity stimulation (F(1, 424) = 7.96, *p* = 0.005). Pain ratings differed across stimulation sites (F(3, 419) = 2.67, *p* = 0.047), with arm stimulation receiving higher ratings than trigeminal stimulation. The intensity effect was consistent across stimulation sites (intensity × stimulation site interaction: F(3, 419) = 0.50, *p* = 0.68)). Stimulation intensities did not differ significantly between male and female participants (median 1.78 vs. 0.97 mA; Wilcoxon rank-sum, W = 224, *p* = 0.112; Hedges’ g = 0.69). Adding sex to the linear mixed model of pain ratings revealed no main effect of sex (F(1, 27.0) = 0.53, *p* = 0.47) and no interaction of sex with intensity (F(1, 425.0) = 1.74, *p* = 0.19) or stimulation site (F(3, 419.0) = 0.83, *p* = 0.48).

**Table 1:** Descriptive statistics of pain ratings by condition and intensity

Condition	Intensity	Mean	SD	*n*
V1	Low	36.2	22.5	55
V1	High	42.0	25.0	58
V2	Low	40.1	21.0	55
V2	High	46.7	23.6	58
V3	Low	41.8	23.2	55
V3	High	42.4	24.3	58
Arm	Low	44.0	21.9	55
Arm	High	49.2	22.8	58

Pain ratings on a 0–100 visual analogue scale. Sessions with a rating of 1 (no perceived sensation) were excluded

### Functional neuroimaging data at the brainstem level

**Fig. 2:**
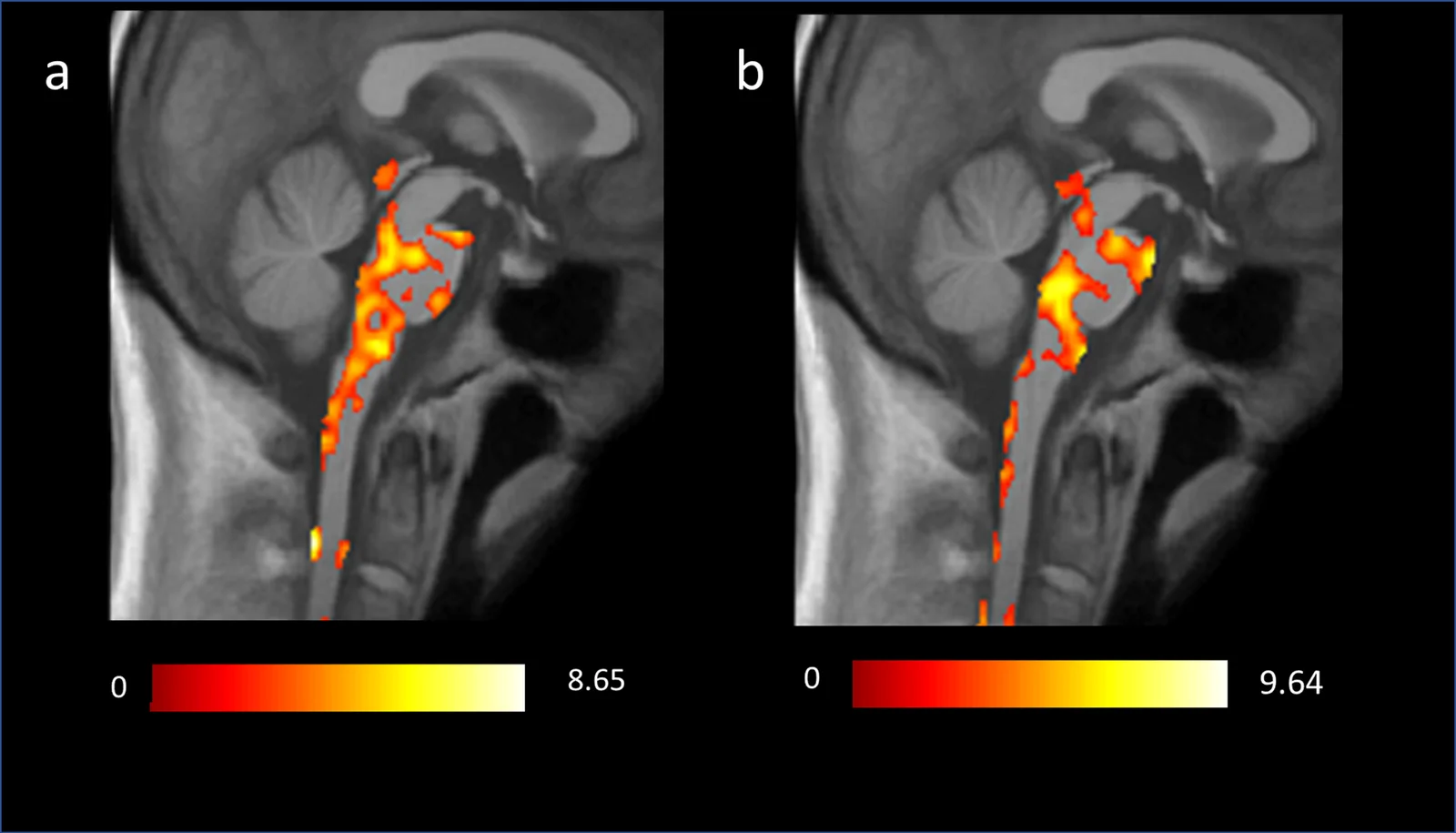
Functional representations of the three trigeminal branches in the brainstem after using unweighted ANOVA (**a**) and rating-weighted ANOVA (**b**). Results are presented for a statistical threshold of T > 3 (*n* = 30, df = 87)

All three trigeminal branches showed significant activation within the spinal trigeminal nucleus at five previously reported levels along the brainstem and upper spinal cord at a statistical threshold of small-volume corrected pFWE = 0.05 ((Fig. 2a, Supplemental Table S1) using previously published coordinates as sphere (Table 2 in [4]).

**Table 2:** Somatotopy in the brainstem below the pons, sorted along the z-axis. Small-volume corrected, 6 mm sphere. Rating-weighted second level analysis (*n* = 30)

Height level of cluster	Cluster extent	T-value	Coordinates (xyz, mm in MNI space)	Reference coordinates
**V1**				
Below pons	212	5.87*	-5, -40, -57	0, -43, -56
Lower cerebellum	14	3.54	0, -43, -69	0, -43, -66
C1	18	3.75*	1, -51, -75	0, -46, -78
C2	211	6.12*	-4, -52, -87	0, -50, -84
C2/C3	28	5.00*	-2, -52, -88	0, -50, -93
**V2**				
Below pons	251	5.16*	-1, -48, -55	0, -44, -56
Lower cerebellum	13	3.62	-2, -51, -70	0, -46, -66
C1	14	3.16	-4, -55, -81	0, -52, -78
C2	38	4.35*	1, -51, -87	0, -47, -84
C2/C3	30	3.65*	2, -51, -89	0, -47, -93
**V3**				
Below pons	54	5.39*	-5, -41, -55	0, -44, -56
Lower cerebellum	2**	3.17	-1, -51, -70	0, -48, -66
C1	21	3.96	-1, -51, -77	0, -46, -78
C2	61	4.57*	-2, -53, -81	0, -49, -84
C2/C3	13	3.73	1, -55, -88	0, -52, -93
**V1 + V2+V3**				
Below pons	277	6.33*	-5, -40, -56	0, -43, -56
Lower cerebellum	7**	3.87*	-1, -51, -69	0, -46, -66
C1	28	4.38*	2, -51, -76	0, -46, -78
C2	241	5.07*	-3, -53, -88	0, -50, -84
C2/C3	30	4.74*	-1, -53, -88	0, -50, -93

*pFWE-corrected, ** <10 cluster

The rating-weighted analysis likewise replicated the five previously reported brainstem representations across all trigeminal branches, indicating that the brainstem activation patterns were not confounded by stimulus intensity. Significant clusters of activation for each trigeminal branches were summarized in Fig. 2b and Table 2. Exploratory analysis at the thresholds (T > 3, uncorrected) identified activation at the level of the lower cerebellum for all three branches and at C1 for V2 and V3. Peak activation coordinates were in close proximity to previously published reference coordinates [4]. We could further identify differences for an intensity matched analysis between the representations for stimulation of V1 and the arm for the two rostral brainstem representations (Supplemental Table S2).

### Somatotopy of the trigeminal branches in the insula, primary somatosensory area (S1), S2/parietal operculum and anterior cingulate cortex

We characterized the somatotopic arrangement after matching perceived intensity across branches using a winner-take-all approach.

#### Insula

**Fig. 3:**
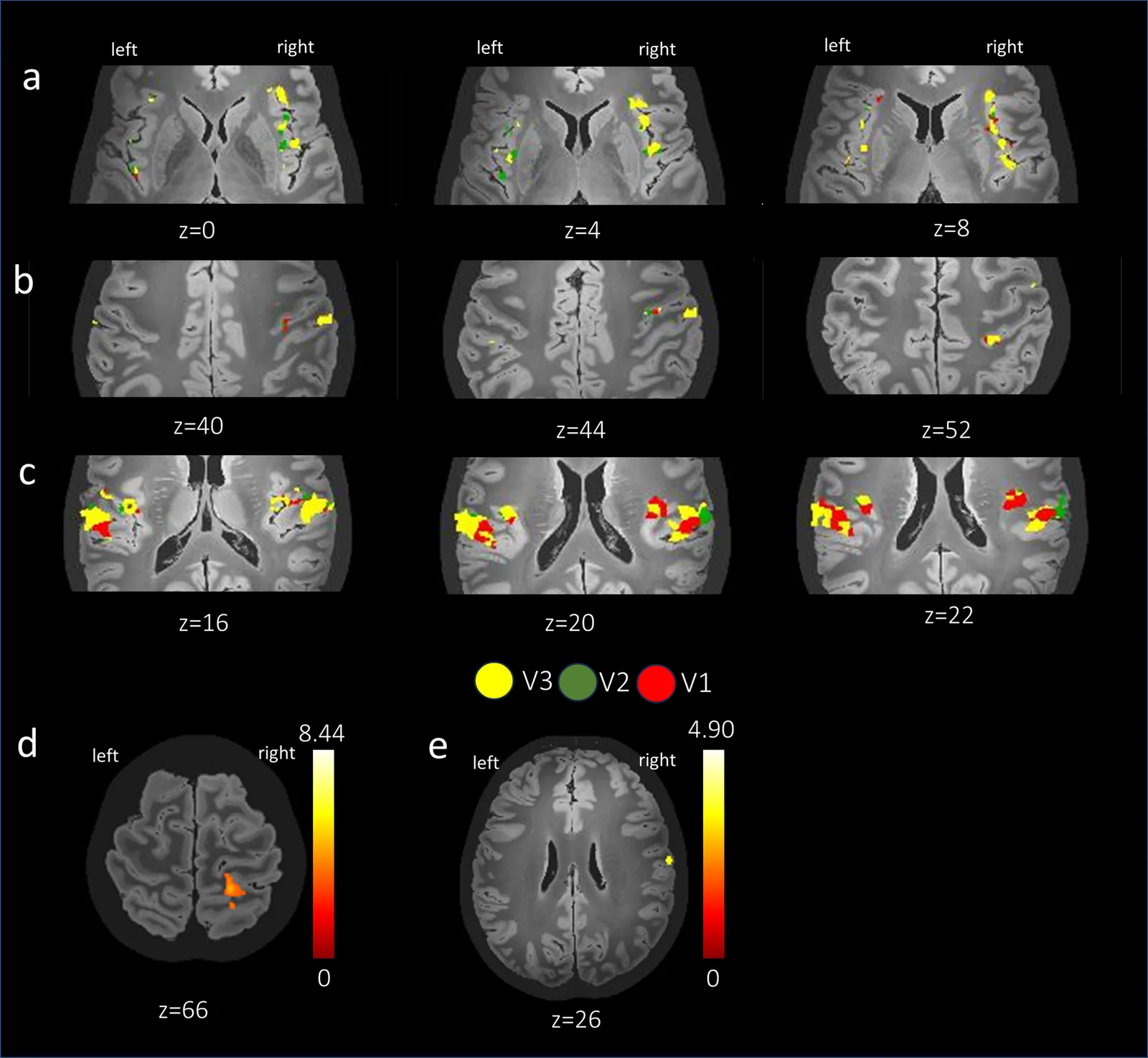
Somatotopic representations of the trigeminal branches (V1, V2, V3) in the insula, primary somatosensory cortex (S1) and S2/parietal operculum using a winner-take-all approach (**a**, **b**, **c**). Activations were predominantly contralateral; ipsilateral activation was observed mainly in S2/parietal operculum, consistent with the known bilateral representation of nociceptive input in this region. Functional activation of the hand (**d**) and face area (**e**) of the primary somatosensory cortex after contrasting arm to trigeminal stimulation and trigeminal to arm stimulation, respectively

In the insula, V3 dominated suprathreshold voxels, followed by V2, with V1 contributing the smallest share (Fig. 3a). V3 dominance was most pronounced in the anterior insula, with smaller but comparable contributions in the intermediate and posterior regions. The overall supra-threshold distribution was contralateral to the side of stimulation especially for V2 and V3, with the strongest contralateral preference in the anterior insula (Id6, Id7) (Table 3).

**Table 3:** Somatotopy in the insula (matched ANOVA, *n* = 23)

Region	Side	V1	V2	V3	Sub-thr.	Total
***Posterior insula***						
Ig1	L	0	0	9	1061	1070
Ig1	R	0	2	0	874	876
Ig2	L	2	3	1	1079	1085
Ig2	R	0	7	51	1206	1264
Ig3	L	45	0	295	487	827
Ig3	R	72	0	340	366	778
**Subtotal posterior**		**119**	**12**	**696**	**5073**	**5900**
***Intermediate insula***						
Id2	L	16	82	55	755	908
Id2	R	0	5	113	700	818
Id3	L	14	15	225	1248	1502
Id3	R	0	32	330	1635	1997
**Subtotal intermediate**		**30**	**134**	**723**	**4338**	**5225**
***Anterior insula***						
Id4	L	8	30	82	910	1030
Id4	R	8	11	198	1078	1295
Id5	L	3	18	75	1132	1228
Id5	R	0	62	192	1270	1524
Id6	L	4	117	248	3993	4362
Id6	R	78	230	789	2706	3803
Id7	L	11	3	12	1720	1746
Id7	R	0	43	593	1273	1909
**Subtotal anterior**		**112**	**514**	**2189**	**14,082**	**16,897**
**Total**		**261**	**660**	**3608**	**23,493**	**28,022**

Number of voxels assigned to each trigeminal branch (V1, V2, V3) within Jülich-Brain Atlas v3.0 insular subregions (probability threshold 25%). At each voxel above the FWE-corrected threshold (*p* < 0.05, peak-level), the branch with the highest T-value was assigned as dominant. Sub-thr. = sub-threshold voxels (within mask but below FWE threshold, T = 4.54). L = left hemisphere; R = right hemisphere

#### S1 and S2/parietal operculum

The analysis revealed contrasting inter-hemispheric patterns between primary somatosensory cortex and S2/parietal operculum. In S1 (Fig. 3b) suprathreshold voxels were strongly contralateral (right) for all three trigeminal branches (Table 4). In S2/parietal operculum (Fig. 3c), suprathreshold voxel counts were essentially symmetric across hemispheres and V3 was the dominant branch bilaterally (Table 4).

**Table 4:** Winner-take-all somatotopy in primary and secondary somatosensory and cingulate cortices (matched ANOVA, *n* = 23)

Region	Side	V1	V2	V3	Sub-thr.	Total
***Primary somatosensory cortex (S1)***						
Area 3a	L	0	3	9	3834	3846
Area 3a	R	54	18	0	2492	2564
Area 3b	L	0	1	173	7712	7886
Area 3b	R	67	65	283	9738	10,153
Area 1	L	0	0	9	5641	5650
Area 1	R	1	0	382	6254	6637
Area 2	L	0	0	7	5375	5382
Area 2	R	126	3	604	6113	6846
**Total S1**		**248**	**90**	**1467**	**47,159**	**48,964**
***S2/Parietal operculum***						
OP1	L	765	89	1372	2246	4472
OP1	R	443	373	1485	1614	3915
OP2	L	68	0	11	823	902
OP2	R	106	0	53	860	1019
OP3	L	103	47	403	794	1347
OP3	R	324	11	276	668	1279
OP4	L	247	258	502	1689	2696
OP4	R	15	308	954	1013	2290
**Total S2/parietal operculum**		**2071**	**1086**	**5056**	**9707**	**17,920**
***Anterior cingulate cortex (ACC)***						
Area 25	L	0	0	0	889	889
Area 25	R	0	0	13	697	710
Area 33	L	0	0	0	3367	3367
Area 33	R	0	0	2	3593	3595
s32	L	0	0	0	2315	2315
s32	R	0	0	0	2245	2245
p32	L	1	0	0	8953	8954
p32	R	0	0	14	8853	8867
p24ab	L	0	0	0	2403	2403
p24ab	R	0	0	0	2408	2408
p24c	L	0	0	0	2704	2704
p24c	R	0	0	0	3885	3885
s24	L	0	0	0	1896	1896
s24	R	0	0	0	1951	1951
**Total ACC**		**1**	**0**	**29**	**46,159**	**46,189**

Number of voxels assigned to each trigeminal branch (V1, V2, V3) within Jülich-Brain Atlas v3.0 subregions; probability threshold 25%. At each voxel above the FWE-corrected threshold (*p* < 0.05, peak-level), the branch with the highest T-value was assigned as dominant. Small-volume correction was computed separately for the combined S1, S2/parietal operculum, and ACC masks. Sub-thr. = sub-threshold voxels (within mask but below FWE threshold, for S1 T = 4.72, S2/parietal operculum T = 4.41, ACC T = 4.68). L = left hemisphere; R = right hemisphere

#### Anterior cingulate cortex

In contrast to the somatosensory cortices, the anterior cingulate cortex showed no meaningful trigeminal somatotopy (Table 4).

Corresponding results for the full cohort using pooled ANOVA are shown in Supplemental Table S3 and followed the same overall pattern, with larger suprathreshold extents across regions.

At the cortical level, arm versus trigeminal stimulation yielded significant activation in the contralateral (right) sensory arm area (Fig. 3d), while the reverse contrast revealed activation in the contralateral face representation of S1 (Fig. 3e).

### Functional connectivity within the brainstem

**Fig. 4:**
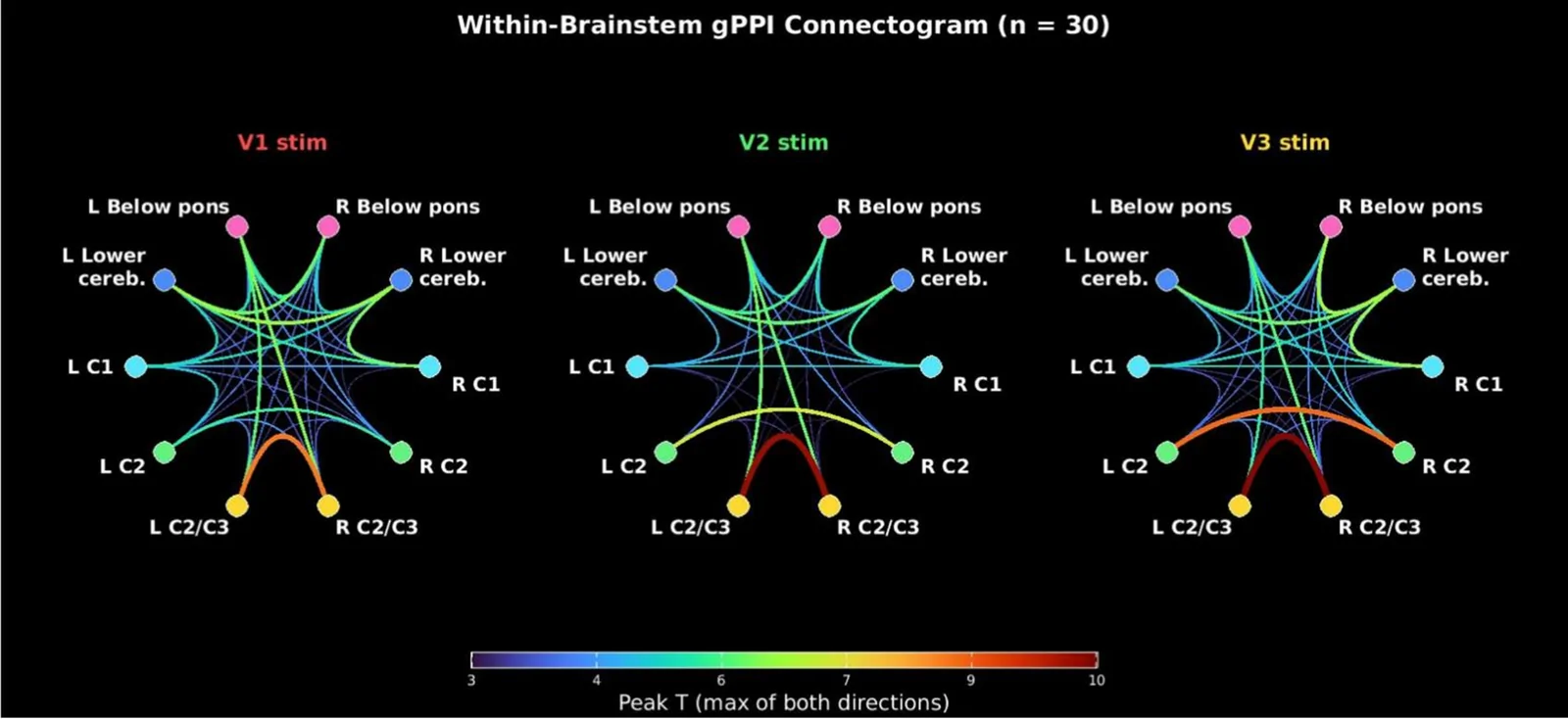
Within-brainstem functional connectivity across five levels of the spinal trigeminal nucleus. Commissural connectivity between homologous left and right seeds and vertical connectivity between levels are shown. gPPI, generalized psychophysiologic interaction

Brainstem areas activated during nociception at five different levels demonstrated strong functional connectivity between each other and to their contralateral counterparts, with 44 of 45 seed pairs reached peak T > 3 across all three stimulation branches (Fig. 4). The strongest connections were always between the left and right sides of the same level, with the pair at the C2/C3 level reaching T-values of ≈ 8.30–9.92 (Supplemental Table S4). The same left-right pattern was present at the other levels, but weaker (T ≈ 4.2–6.4). Strong connections have also been detected between the left below-pons level and bilateral C2/C3 levels (T ≈ 5.6–6.2). Coupling between non-matching levels was lower but still mostly supra-threshold (T ≈ 3.0–5.7). This overall pattern was greatly independent of which trigeminal branch was stimulated as the interaction effect was not significant (all F(2,116) ≤ 5.67, threshold F = 8.00, α = 0.05/90).

### Functional connectivity between brainstem seeds and cortex

**Fig. 5:**
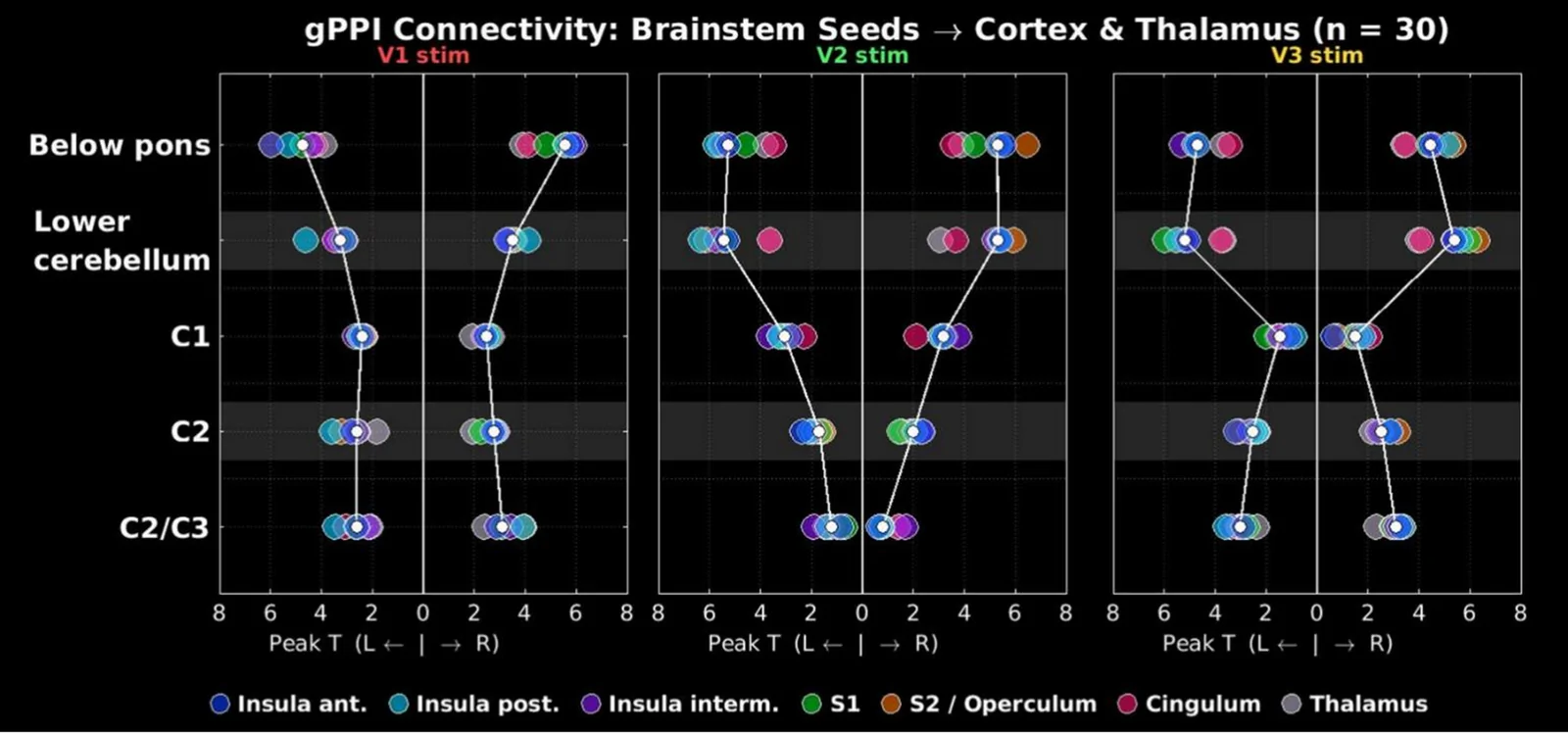
Functional connectivity between brainstem seeds and pre-registered cortical regions. Each dot represents the peak T-value of a seed-target connection. Left-hemispheric targets are plotted on the negative x-axis, right-hemispheric targets on the positive x-axis. gPPI, generalized psychophysiologic interaction

**Fig. 6:**
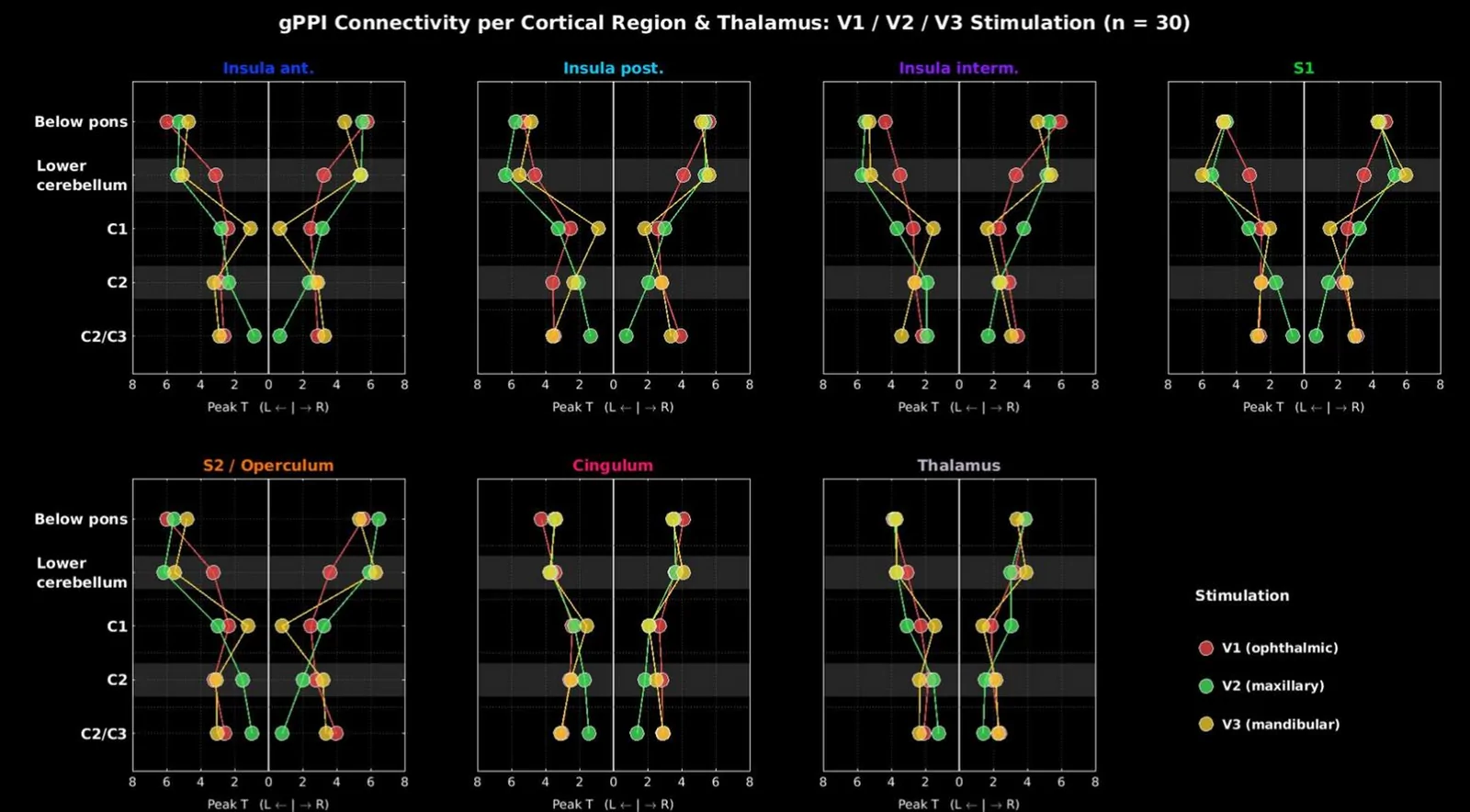
Functional connectivity between brainstem seeds and pre-registered cortical regions. Data are shown for each cortical region and thalamus, separately for stimulation of the three trigeminal branches. Each dot represents the peak T-value of a seed-target connection. Left-hemispheric targets are plotted on the negative x-axis, right-hemispheric targets on the positive x-axis. gPPI, generalized psychophysiologic interaction

Significant functional connectivity between brainstem seeds and cortical targets across all three trigeminal branches was identified (Table 5; Fig. 5). Connectivity was strongest from the most rostral seed (below pons) and decreased toward more caudal levels. A two-factor ANOVA (stimulation branch × brainstem level) confirmed this rostrocaudal gradient: the main effect of brainstem level survived Bonferroni correction across all insular subdivisions bilaterally (Id2–Id7, Ig1–Ig3), the primary somatosensory cortex (Areas 3a, 3b, 1, 2), the S2/parietal operculum (OP1–OP4), several cingulate subregions (p24c, p24ab, p32 bilaterally; Area 25 right), and thalamus. The main effect of stimulation branch survived correction in the subgenual cingulate cortex (s24 bilaterally) and thalamus only (Table 5; Fig. 6). The interaction between stimulation branch and brainstem level survived correction bilaterally in the anterior insula (Id4–Id7), intermediate insula (Id2 right, Id3 bilaterally), posterior insula (Ig1 left, Ig3 bilaterally), primary somatosensory cortex (Area 1 bilaterally), S2/parietal operculum (OP1 and OP4 bilaterally; OP2 and OP3 left), and thalamus bilaterally, indicating that the rostrocaudal connectivity gradient was modulated in a branch-specific manner (Table 5).

**Table 5:** Functional connectivity between brainstem nuclei and cortical regions: two-factor ANOVA (stimulation branch × brainstem level)

Target ROI	Branch (F01)				Level (F02)			Interaction (F03)	
	F		pFWE		F		pFWE	F	pFWE
***Insula anterior***									
Id6 (ant) (R)	3.63		0.106		10.95		< 0.001^**^	3.62	0.002^**^
Id6 (ant) (L)	2.75		0.233		10.64		< 0.001^**^	4.74	< 0.001^**^
Id7 (ant ventral) (R)	3.16		0.178		9.65		< 0.001^**^	5.19	< 0.001^**^
Id7 (ant ventral) (L)	4.06		0.078		7.08		< 0.001^**^	4.37	< 0.001^**^
Id4 (R)	3.67		0.092		10.90		< 0.001^**^	3.72	0.001^**^
Id4 (L)	2.05		0.377		8.34		< 0.001^**^	4.24	< 0.001^**^
Id5 (R)	4.33		0.061		10.47		< 0.001^**^	4.18	< 0.001^**^
Id5 (L)	–		–		9.97		< 0.001^**^	3.68	0.002^**^
***Insula posterior***									
Ig1 (deep) (R)	–		–		10.16		< 0.001^**^	–	–
Ig1 (deep) (L)	3.91		0.089		9.67		< 0.001^**^	3.46	0.003^**^
Ig2 (post) (R)	–		–		9.60		< 0.001^**^	2.30	0.071
Ig2 (post) (L)	3.91		0.073		9.86		< 0.001^**^	2.46	0.047^*^
Ig3 (R)	2.78		0.206		9.96		< 0.001^**^	3.07	0.008^**^
Ig3 (L)	–		–		10.16		< 0.001^**^	3.63	0.002^**^
***Insula intermediate***									
Id2 (R)	2.87		0.210		6.04		< 0.001^**^	3.72	0.001^**^
Id2 (L)	3.56		0.113		10.46		< 0.001^**^	2.77	0.022^*^
Id3 (R)	4.86		0.033^*^		10.05		< 0.001^**^	3.46	0.003^**^
Id3 (L)	2.26		0.351		8.78		< 0.001^**^	3.32	0.005^**^
***S1***									
Area 3a (R)	2.71		0.262		12.56		< 0.001^**^	3.07	0.010^*^
Area 3a (L)	2.94		0.217		16.22		< 0.001^**^	2.61	0.038^*^
Area 3b (R)	6.41		0.007^*^		9.93		< 0.001^**^	2.51	0.040^*^
Area 3b (L)	–		–		9.34		< 0.001^**^	–	–
Area 1 (R)	5.40		0.020^*^		9.67		< 0.001^**^	3.32	0.004^**^
Area 1 (L)	5.50		0.018^*^		9.80		< 0.001^**^	3.27	0.005^**^
Area 2 (R)	3.38		0.121		8.07		< 0.001^**^	2.34	0.065
Area 2 (L)	6.31		0.007^*^		8.83		< 0.001^**^	2.37	0.061
***S2/Parietal operculum***									
OP1 [SII] (R)	3.24		0.165		10.89		< 0.001^**^	3.84	0.001^**^
OP1 [SII] (L)	–		–		10.24		< 0.001^**^	3.99	0.001^**^
OP2 [PIVC] (R)	2.67		0.250		12.59		< 0.001^**^	3.16	0.007^*^
OP2 [PIVC] (L)	2.54		0.278		9.82		< 0.001^**^	3.43	0.003^**^
OP3 [VS] (R)	2.78		0.206		10.90		< 0.001^**^	3.10	0.008^*^
OP3 [VS] (L)	2.70		0.221		10.16		< 0.001^**^	3.63	0.002^**^
OP4 [PV] (R)	–		–		11.22		< 0.001^**^	4.10	< 0.001^**^
OP4 [PV] (L)	–		–		12.01		< 0.001^**^	3.96	0.001^**^
***Cingulum***									
Area 33 (ACC) (R)	3.19		0.173		4.42		0.007^*^	2.84	0.020^*^
Area 33 (ACC) (L)	3.77		0.102		3.65		0.028^*^	2.81	0.021^*^
p24c (MCC) (R)	5.28		0.020^*^		6.15		< 0.001^**^	2.89	0.014^*^
p24c (MCC) (L)	3.96		0.071		7.93		< 0.001^**^	2.64	0.029^*^
p24ab (R)	6.16		0.009^*^		5.82		0.001^**^	2.53	0.040^*^
p24ab (L)	6.96		0.004^*^		7.20		< 0.001^**^	2.41	0.054
s24 (R)	7.42		0.003^**^		3.22		0.046^*^	2.60	0.033^*^
s24 (L)	8.34		0.001^**^		3.76		0.019^*^	–	–
p32 (R)	5.88		0.011^*^		7.22		< 0.001^**^	2.31	0.070
p32 (L)	3.94		0.071		7.93		< 0.001^**^	2.35	0.063
s32 (R)	3.98		0.069		2.54		0.135	2.08	0.126
s32 (L)	4.09		0.062		4.22		0.009^*^	2.83	0.017^*^
Area 25 (subgen) (R)	6.33		0.008^*^		4.99		0.003^**^	2.34	0.071
Area 25 (subgen) (L)	5.12		0.026^*^		4.14		0.011^*^	3.23	0.006^*^
***Thalamus***									
Thalamus (R)		6.62		<0.001**		12.01	<0.001**	3.18	<0.001**
Thalamus (L)		4.57		<0.001**		10.85	<0.001**	3.97	<0.001**

SVC: 6 mm sphere around Jülich-Brain Atlas v3.0 and Morel thalamus Atlas centroids. F = peak F-value; ^*^pFWE < 0.05 (peak-level); ^**^pFWE surviving within-region Bonferroni correction, with α = 0.05/n applied separately within each a priori defined anatomical region, treating left and right ROIs as separate tests (Insula anterior *n* = 8, α = 0.00625; Insula posterior *n* = 6, α = 0.00833; Insula intermediate *n* = 4, α = 0.0125; S1 *n* = 8, α = 0.00625; S2/parietal operculum *n* = 8, α = 0.00625; Cingulum *n* = 14, α = 0.00357, Thalamus *n* = 2, α = 0.025). – = F < 2 or not significant

## Discussion

The present study replicated our previous finding that functional brainstem representations across the three trigeminal branches are largely overlapping and bilaterally organized [4] and extended this observation to multiple cortical regions involved in trigeminal pain processing. The main finding is a bilateral representation of trigeminal pain, present right from the brainstem level and consistent across nearly all subcortical and higher cortical areas. The only notable exception was the primary somatosensory cortex (S1, for face and arm) and insula (for face), where activation remained predominantly contralateral to the stimulation site. Together, these findings suggest that trigeminal nociception engages a broad, bilaterally distributed brainstem and cortical network, while simultaneously reflecting strong cross-midline connectivity at the brainstem level and region-specific cortical connectivity patterns depending on the brainstem structure involved.

The bilateral cortical activation observed in the present study is anatomically supported by the extensive connectivity of the STN with brainstem nuclei involved in pain modulation including the periaqueductal gray [32], parabrachial nucleus [33], rostral ventromedial medulla [34], and locus coeruleus [35]), as well as ascending projections to the thalamus [36, 37] and hypothalamus [38]. Many of these pathways are organized bilaterally, providing multiple routes through which unilateral trigeminal nociceptive input can engage bilateral cortical networks [39–43]. Within the STN, previous anatomical studies have identified fusiform and multipolar neurons that form ipsilateral ascending and descending intra-trigeminal pathways as well as commissural intra-trigeminal connections [44–46]. This is consistent with our finding of strong within-brainstem functional connectivity, both between bilateral seeds at the same rostrocaudal level and between seeds at different levels [45]. The bilateral cortical activation in S2/parietal operculum observed in our study, which is consistent with prior reports following nociceptive trigeminal stimulation [39–41, 43], may in part reflect such commissural brainstem connections, although interhemispheric cortical pathways may contribute as well [47].

In contrast, contralateral activation in S1 and insula has been reported after trigeminal nociceptive stimulation, particularly for the V2 and V3 branches [48–50], a pattern that was also observed in the present study. Accurate localization of noxious stimulation is necessary to identify its source and to adapt the behavioral response to it. This requirement may be particularly relevant for trigeminal nociception: the face contains multiple vital structures within a small area, and protective responses must be precisely directed at the affected region. Consistent with this, trigeminal pain sensitizes more strongly compared to extratrigeminal pain [51]. Taken together, the contralateral predominance of activation we observed in the insula and S1 is consistent with a system optimized for spatial localization of trigeminal pain. The observed V2 and V3 dominance in cortical somatotopy may reflect differences in peripheral innervation density and central representation across trigeminal divisions [52–54].

gPPI functional connectivity analyses are inherently non-directional. The functional connectivity observed in our study therefore could reflect both, trigemino-thalamo-cortical and descending cortico-trigeminal pathways, the latter of which being characterized in animal studies [55–57]. Anatomical evidence, however, suggests that these two systems within the brainstem are organized along opposing rostrocaudal gradients: corticotrigeminal descending projections terminate most densely in the pars caudalis [57], whereas trigemino-thalamo-cortical ascending projections are most pronounced rostrally [58–60]. In our data, the strongest functional connectivity to the cortex was observed at the most rostral levels, consistent with ascending trigemino-thalamic-cortical rather than descending cortico-trigeminal projections driving the observed connectivity. Further supporting this interpretation, thalamic regions showed concordant functional connectivity with both cortical regions and trigeminal subnuclei — a pattern expected if the observed connectivity is mediated by ascending trigemino-thalamic-cortical transmission, given that the thalamus is not part of the descending cortico-trigeminal pathway [57].

The well-known somatotopic organization of trigeminal divisions within the brainstem provides a plausible anatomical substrate for the branch-specific connectivity patterns observed in the present study. Because these neuronal populations maintain somatotopically organized ascending projections [54, 61], the coupling between a given brainstem level and cortical targets is expected to vary as a function of the stimulated branch. The absence of a significant branch effect on cortical activation possibly reflects the convergence of all three trigeminal divisions onto shared cortical targets, resulting in comparable net nociceptive cortical activation [49, 62]. The presence of branch-specific trigemino-cortical connectivity, however, suggests that the pathways mediating this convergence remain anatomically segregated.

The present study demonstrates that trigeminal nociceptive processing engages a broadly distributed brainstem and cortical network, primarily bilateral in S2 but with contralateral predominance in S1 and insula. We further identified branch-specific functional connectivity between the STN and cortical regions, with a rostral brainstem predominance consistent with the known distribution of ascending trigemino-thalamo-cortical projections. However, methodological considerations should be noted. Given that the chosen neuroimaging sequence was not exclusively brainstem specific, the rostral brainstem predominance of cortical connectivity could in principle reflect lower spatial resolution and higher susceptibility artifacts in the caudal brainstem. Nonetheless, this technical effect cannot account for the branch-specific modulation observed, indicating that the gradient reflects genuine connectivity differences. Similarly, the 4 mm smoothing kernel applied to the group-level contrast images may introduce partial-volume mixing between adjacent left and right brainstem voxels, particularly at more caudal levels where the cross-section narrows. The lateralized within-brainstem connectivity is therefore reported descriptively, and the anatomical interpretation of commissural connectivity rests on independent anatomical evidence rather than on the observed effect magnitudes alone. Furthermore, the non-directional nature of the connectivity analyses precludes a definite differentiation between ascending and descending pathways. Future studies should investigate whether these trigeminal connectivity patterns are altered in primary headache disorders such as migraine or cluster headache, which may provide insights into pain-related mechanisms at multiple levels of the neuraxis.

## Supplementary Information

Below is the link to the electronic supplementary material.


Supplementary Material 1


## Data Availability

Access to anonymized data and access to the code are available to qualified investigators on request to the corresponding author.
