# Supplementary material for: Trigeminal nociceptive somatotopy across brainstem and cortex and their functional interactions
Source: J Headache Pain. 2026 Aug 31;27(1):218. doi: 10.1186/s10194-026-02476-y (PMC13528110; doi:10.1186/s10194-026-02476-y)
Supplement: Supplementary file 1 [file 10194_2026_2476_MOESM1_ESM.docx]

**Supplemental Table S1. Unweighted analysis: Somatotopy in the brainstem below the pons, sorted along the z-axis. Small-volume corrected, 6 mm sphere.**

| Height level of cluster | Cluster extent | T-value | Coordinates (xyz, mm in MNI) space) | Reference coordinates |
| --- | --- | --- | --- | --- |
| **V1** | | | | |
| Below pons | 289 | 5.53* | 1, −38, −53 | 0, −43, −56 |
| Lower cerebellum | 82 | 4.27* | 1, −45, −61 | 0, −43, −66 |
| C1 | 8 | 4.03* | 4, −50, −80 | 0, −46, −78 |
| C2 | 112 | 4.63* | 5, −51, −81 | 0, −50, −84 |
| C2/C3 | – | – | – | 0, −50, −93 |
| **V2** | | | | |
| Below pons | 42 | 3.96* | 2, −43, −60 | 0, −44, −56 |
| Lower cerebellum | 47 | 4.26* | −5, −45, −63 | 0, −46, −66 |
| C1 | 18 | 3.68* | 5, −51, −79 | 0, −52, −78 |
| C2 | 3 | 3.45* | −4, −43, −82 | 0, −47, −84 |
| C2/C3 | – | – | – | 0, −47, −93 |
| **V3** | | | | |
| Below pons | 65 | 4.74* | 1, −39, −53 | 0, −44, −56 |
| Lower cerebellum | 67 | 4.13* | 2, −52, −70 | 0, −48, −66 |
| C1 | 35 | 4.34* | 4, −50, −80** | 0, −46, −78 |
| C2 | 35 | 4.34* | 4, −50, −80** | 0, −49, −84 |
| C2/C3 | – | – | – | 0, −52, −93 |
| **V1+V2+V3** | | | | |
| Below pons | 366 | 7.03* | 1, -38, -53 | 0, -43, -56 |
| Lower cerebellum | 405 | 5.48* | 2, -44, -61 | 0, -46, -66 |
| C1 | 93 | 6.21* | 4, -50, -80 | 0, -46, -78 |
| C2 | 131 | 6.61* | 5, -50, -81 | 0, -50, -84 |
| C2/C3 | - | - | - | 0, -50, -93 |

*pFWE<0.05. **Where reference coordinates of adjacent levels are separated by less than the SVC sphere diameter, spheres overlap; in such cases, a single peak may appear in two levels.

**Supplemental Table S2. Trigeminal versus arm brainstem activation across analyses.**

| **Analysis** | **Contrast** | **Level** | **k** | **T** | **pFWE** | **Peak MNI** |
| --- | --- | --- | --- | --- | --- | --- |
| **Trigeminal > Arm** | | | | | | |
| **Matched Hi>Lo** | V1 > Arm | Below pons | 56 | 3.67 | .001* | −2, −45, −57 |
|  |  | Lower cerebellum | 5 | 3.18 | .005* | −2, −46, −61 |
|  | V2 > Arm | Below pons | 30 | 3.51 | .002* | −2, −43, −53 |
|  | V3 > Arm | all levels | – | – | – | – |
|  | Trig > Arm | Below pons | 42 | 3.63 | .001* | −2, −44, −54 |
| **Arm > Trigeminal** | | | | | | |
| **Matched Hi>Lo** | Arm > V1 | C1 | 4 | 3.14 | .006* | −1, −42, −82 |
|  | Arm > V2 | C1 | 7 | 3.16 | .005* | −5, −47, −80 |
|  | Arm > V3 | all levels | – | – | – | – |
|  | Arm > Trig | C1 | 26 | 3.17 | .005* | −1, −43, −83 |
|  |  | C2 | 5 | 3.12 | .006* | −4, −47, −82 |

*Small-volume correction: 6 mm sphere around reference coordinates from Mehnert et al. (2023): Below pons [0, −43, −56], Lower cerebellum [0, −46, −66], C1 [0, −46, −78], C2 [0, −50, −84], C2/C3 [0, −50, −93]. k = cluster extent in voxels; T = peak T-value; *pFWE < 0.05 (peak-level). – = no suprathreshold voxels (T > 3). Intensity-contrast ANOVA: |V1 − Arm| ≤ 15 (67 sessions, 27 participants).*

**Supplemental Table S3. Winner-take-all somatotopy in the insula, and somatosensory cortices (unweighted ANOVA, n = 30).**

| **Region** | **Side** | **V1** | **V2** | **V3** | **Sub-thr.** | **Total** |
| --- | --- | --- | --- | --- | --- | --- |
| Posterior insula | | | | | | |
| Ig1 | L | 0 | 0 | 101 | 969 | 1070 |
| Ig1 | R | 5 | 0 | 4 | 867 | 876 |
| Ig2 | L | 0 | 6 | 555 | 524 | 1085 |
| Ig2 | R | 16 | 34 | 375 | 839 | 1264 |
| Ig3 | L | 13 | 0 | 648 | 166 | 827 |
| Ig3 | R | 113 | 43 | 497 | 125 | 778 |
| **Subtotal posterior** |  | **147** | **83** | **2180** | **3490** | **5900** |
| Intermediate insula | | | | | | |
| Id2 | L | 170 | 204 | 392 | 142 | 908 |
| Id2 | R | 117 | 108 | 527 | 66 | 818 |
| Id3 | L | 66 | 39 | 1239 | 158 | 1502 |
| Id3 | R | 44 | 130 | 1445 | 378 | 1997 |
| **Subtotal intermediate** |  | **397** | **481** | **3603** | **744** | **5225** |
| Anterior insula | | | | | | |
| Id4 | L | 138 | 59 | 500 | 333 | 1030 |
| Id4 | R | 143 | 149 | 386 | 617 | 1295 |
| Id5 | L | 392 | 253 | 403 | 180 | 1228 |
| Id5 | R | 122 | 608 | 435 | 359 | 1524 |
| Id6 | L | 727 | 530 | 1198 | 1907 | 4362 |
| Id6 | R | 203 | 982 | 1883 | 735 | 3803 |
| Id7 | L | 350 | 37 | 469 | 890 | 1746 |
| Id7 | R | 102 | 185 | 1382 | 240 | 1909 |
| **Subtotal anterior** |  | **2177** | **2803** | **6656** | **5261** | **16897** |
| **Subtotal insula** |  | **2721** | **3367** | **12439** | **9495** | **28022** |
| Primary somatosensory cortex (S1) | | | | | | |
| Area 3a | L | 21 | 12 | 22 | 3791 | 3846 |
| Area 3a | R | 60 | 173 | 2 | 2329 | 2564 |
| Area 3b | L | 26 | 11 | 327 | 7522 | 7886 |
| Area 3b | R | 324 | 268 | 315 | 9246 | 10153 |
| Area 1 | L | 10 | 3 | 73 | 5564 | 5650 |
| Area 1 | R | 5 | 128 | 465 | 6039 | 6637 |
| Area 2 | L | 19 | 5 | 64 | 5294 | 5382 |
| Area 2 | R | 544 | 172 | 456 | 5674 | 6846 |
| **Subtotal S1** |  | **1009** | **772** | **1724** | **45459** | **48964** |
| S2/Parietal operculum | | | | | | |
| OP1 | L | 735 | 380 | 2301 | 1056 | 4472 |
| OP1 | R | 863 | 864 | 1612 | 576 | 3915 |
| OP2 | L | 15 | 0 | 159 | 728 | 902 |
| OP2 | R | 174 | 8 | 126 | 711 | 1019 |
| OP3 | L | 97 | 163 | 689 | 398 | 1347 |
| OP3 | R | 390 | 82 | 649 | 158 | 1279 |
| OP4 | L | 244 | 455 | 1258 | 739 | 2696 |
| OP4 | R | 169 | 681 | 856 | 584 | 2290 |
| **Subtotal S2/operculum** |  | **2687** | **2633** | **7650** | **4950** | **17920** |
| Anterior cingulate cortex (ACC) | | | | | | |
| Area 25 | L | 0 | 0 | 0 | 889 | 889 |
| Area 25 | R | 0 | 0 | 3 | 707 | 710 |
| Area 33 | L | 0 | 0 | 3 | 3356 | 3367 |
| Area 33 | R | 0 | 0 | 50 | 3545 | 3595 |
| s32 | L | 0 | 0 | 0 | 2315 | 2315 |
| s32 | R | 0 | 0 | 0 | 2245 | 2245 |
| p32 | L | 10 | 1 | 191 | 8752 | 8954 |
| p32 | R | 67 | 0 | 567 | 8233 | 8867 |
| p24ab | L | 0 | 0 | 9 | 2394 | 2403 |
| p24ab | R | 0 | 0 | 288 | 2120 | 2408 |
| p24c | L | 0 | 0 | 29 | 2675 | 2704 |
| p24c | R | 23 | 0 | 121 | 3741 | 3885 |
| s24 | L | 0 | 0 | 0 | 1896 | 1896 |
| S24 | R | 0 | 0 | 0 | 1951 | 1951 |
| **Subtotal ACC** |  | **100** | **9** | **1261** | **44819** | **46189** |

*Number of voxels assigned to each trigeminal branch (V1, V2, V3) — within Jülich-Brain Atlas v3.0 insular subregions (probability threshold 25. At each voxel exceeding the region-specific FWE-corrected SVC threshold (mask-dependent, Insula T > 4.40, S1 T > 4.57, S2/parietal operculum T > 4.27, ACC T >4.53), the branch with the highest T-value was assigned as dominant. Sub-thr. = sub-threshold voxels (within mask but below threshold). L = left hemisphere; R = right hemisphere.*

**Supplemental Table S4. Within-brainstem gPPI peak T-values across all seed-target pairs.**

| **Seed A** | **Seed B** | **V1** | **V2** | **V3** |
| --- | --- | --- | --- | --- |
| L Below pons | R Below pons | 5.70 | 4.94 | 4.24 |
| L Below pons | L Lower cereb. | 5.36 | 4.97 | 4.77 |
| L Below pons | R Lower cereb. | 5.08 | 4.67 | 4.72 |
| L Below pons | L C1 | 3.90 | 2.89 | 3.56 |
| L Below pons | R C1 | 4.10 | 3.25 | 3.74 |
| L Below pons | L C2 | 3.69 | 4.10 | 3.90 |
| L Below pons | R C2 | 4.31 | 4.35 | 4.69 |
| L Below pons | L C2/C3 | 5.91 | 6.04 | 5.59 |
| L Below pons | R C2/C3 | 6.22 | 6.01 | 5.94 |
| R Below pons | L Lower cereb. | 6.04 | 5.75 | 5.13 |
| R Below pons | R Lower cereb. | 5.21 | 5.35 | 6.48 |
| R Below pons | L C1 | 4.01 | 3.80 | 4.26 |
| R Below pons | R C1 | 4.16 | 4.16 | 4.59 |
| R Below pons | L C2 | 4.07 | 3.47 | 3.88 |
| R Below pons | R C2 | 4.31 | 3.74 | 5.03 |
| R Below pons | L C2/C3 | 3.92 | 3.15 | 3.16 |
| R Below pons | R C2/C3 | 3.87 | 3.40 | 4.16 |
| L Lower cereb. | R Lower cereb. | 6.41 | 5.97 | 6.04 |
| L Lower cereb. | L C1 | 5.26 | 4.71 | 4.83 |
| L Lower cereb. | R C1 | 5.42 | 4.78 | 5.12 |
| L Lower cereb. | L C2 | 3.18 | 2.41 | 2.21 |
| L Lower cereb. | R C2 | 3.20 | 2.43 | 2.63 |
| L Lower cereb. | L C2/C3 | 3.44 | 2.41 | 2.96 |
| L Lower cereb. | R C2/C3 | 3.82 | 2.85 | 3.97 |
| R Lower cereb. | L C1 | 5.16 | 4.80 | 5.13 |
| R Lower cereb. | R C1 | 6.14 | 5.48 | 6.29 |
| R Lower cereb. | L C2 | 3.43 | 2.44 | 3.27 |
| R Lower cereb. | R C2 | 3.33 | 2.53 | 4.06 |
| R Lower cereb. | L C2/C3 | 3.41 | 3.08 | 3.30 |
| R Lower cereb. | R C2/C3 | 3.54 | 2.93 | 3.74 |
| L C1 | R C1 | 4.53 | 4.95 | 5.26 |
| L C1 | L C2 | 5.03 | 3.46 | 3.81 |
| L C1 | R C2 | 3.71 | 2.54 | 3.41 |
| L C1 | L C2/C3 | 2.43 | 3.11 | 2.86 |
| L C1 | R C2/C3 | 2.60 | 3.29 | 3.23 |
| R C1 | L C2 | 2.39 | 2.04 | 2.01 |
| R C1 | R C2 | 3.35 | 2.64 | 3.03 |
| R C1 | L C2/C3 | 2.23 | 2.95 | 3.27 |
| R C1 | R C2/C3 | 2.67 | 3.20 | 3.46 |
| L C2 | R C2 | 5.58 | 7.00 | 8.47 |
| L C2 | L C2/C2 | 4.30 | 3.40 | 4.27 |
| L C2 | R C2/C3 | 4.41 | 3.52 | 4.40 |
| R C2 | L C2/C3 | 3.69 | 3.11 | 3.81 |
| R C2 | R C2/C3 | 3.81 | 3.31 | 3.80 |
| L C2/C3 | R C2/C3 | 8.30 | 9.76 | 9.92 |

*Peak T-values for within-brainstem gPPI connectivity from the second-level flexible factorial ANOVA (stimulation branch × brainstem level, lateralized seeds, n = 30). For each seed-target pair, the maximum T-value across both seed→target directions is reported. L = left hemisphere; R = right hemisphere.*
